# Protective Effect and Possible Mechanisms of Astragaloside IV in Animal Models of Diabetic Nephropathy: A Preclinical Systematic Review and Meta-Analysis

**DOI:** 10.3389/fphar.2020.00988

**Published:** 2020-06-30

**Authors:** Hong Wang, Zhuang Zhuang, Yue-Yue Huang, Zhi-Zhi Zhuang, Yi Jin, Han-Yang Ye, Xiao-Ji Lin, Qun Zheng, Yi-Luan Wang

**Affiliations:** Department of Medicine, the Second Affiliated Hospital and Yuying Children’s Hospital of Wenzhou Medical University, Wenzhou, China

**Keywords:** astragaloside IV, rodent models, diabetic nephropathy, meta-analysis, systematic review

## Abstract

Astragaloside IV (AS-IV) has a variety of biological activities and is widely used to treat kidney diseases. We conducted a systematic review of 24 animal studies including 424 animals to evaluate the efficacy of AS-IV for diabetic nephropathy (DN); all current possible mechanisms were summarized. A search strategy was applied to eight databases from inception to June 2020. The CAMARADES 10-item quality checklist and Rev-Man 5.3 software were used to analyze the risks of bias of each study and data regarding outcome measures, respectively. The mean study quality score was 5.4 points (range 3–8 points). Meta-analyses data and comparisons between groups showed that AS-IV significantly slowed the progression of pathological signs in the kidney including glomeruli and tubules, increasing creatinine clearance rate, decreasing blood urea nitrogen, serum creatinine, 24-h urinary neutrophil gelatinase-associated lipocalin and N-acetyl-*β*-D-glucosaminidase, 24-h urinary albumin, 24-h urinary microalbumin and HbA1c. There were no significant differences between experimental and control groups with respect to mortality or levels of alanine aminotransferase and aspartate aminotransferase. In terms of the possible mechanisms of treatment of DN, AS-IV acts through antifibrotic, antioxidant, and antiapoptotic mechanisms, thereby alleviating endoplasmic reticulum stress, inhibiting mitochondrial fission, and increasing autophagic activity. Taken together, our findings suggest that AS-IV is a multifaceted renoprotective candidate drug for DN.

## Introduction

Diabetic nephropathy (DN) is defined as impairment of renal function arising from chronic hyperglycemia. It is among the most severe and common microvascular complications of diabetes mellitus (Saran et al., 2015; Flyvbjerg, 2017). It is characterized by proteinuria, hypertension, and progressive renal insufficiency resulting from compromise of the glomerular filtration barrier, eventually resulting in end-stage renal disease (ESRD) in 30–40% of patients (Bjornstad et al., 2014; Saran et al., 2015; Wang et al., 2016; Du et al., 2017). Management of hypertension and hyperglycemia is the most commonly used approach for treatment of DN. These have been shown to reduce the proportion of patients reaching ESRD (National Kidney Foundation, 2012), although with limited effects (Sharma et al., 2017). Unfortunately, no new therapies that specifically improve progression of DN have been translated into clinical use successfully (Fernandez-Fernandez et al., 2014). For this reason, new therapies are needed urgently to prevent progressive renal failure in these patients.

**Figure 1:**
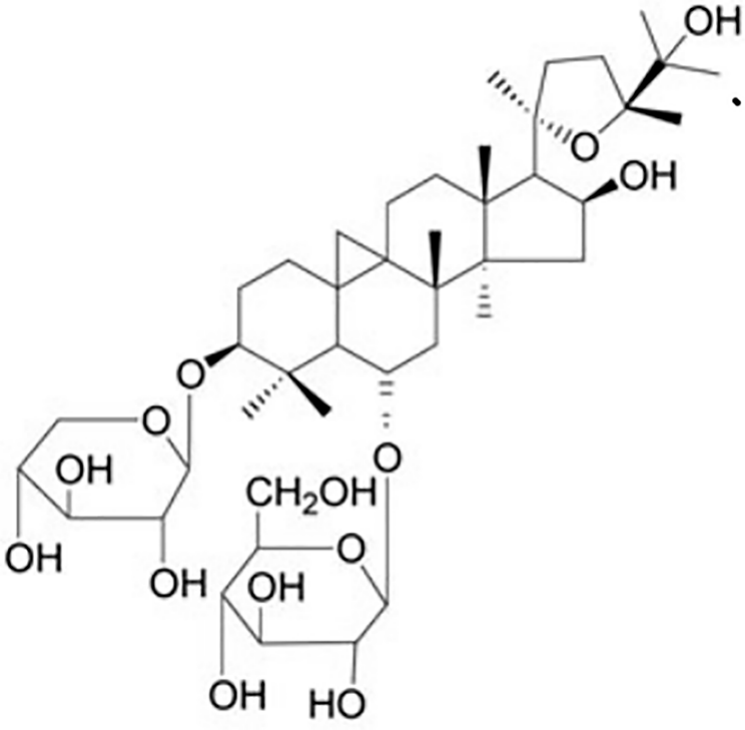
Chemical structures of astragaloside IV.

*Astragalus membranaceus* (Fisch.) Bunge is a perennial herbal plant of the Leguminous family that first appeared in the earliest complete Pharmacopoeia of China (Sheng Nong’s herbal classic work). It was thought to promote Qi according to the theory of traditional Chinese medicine and therefore was widely used for various kidney diseases for thousands of years. Astragaloside IV (AS-IV, C_41_H_68_O_14_, molecular weight = 784, **Figure 1**) is a small molecular saponin that is the active ingredient in *A. membranaceus* (Fisch.) Bunge. Recent studies demonstrated that AS-IV has several pharmacological activities *in vivo* and *in vitro*, including anti-inflammatory, antioxidative, antiapoptotic, antifibrotic, and immunoregulatory functions. AS-IV has also been reported to improve the prognosis of DN in streptozotocin-induced (STZ) diabetic rats *via* inhibition of renal inflammation (Gui et al., 2013a), inhibition of renal oxidative stress (Gui et al., 2013b), attenuation of podocyte apoptosis (Guo et al., 2016), and subsequent delay of DN progression (Sun et al., 2016). Nevertheless, the efficacy of AS-IV for DN has not been evaluated systematically, and the mechanisms have not been summarized comprehensively. Therefore, the aim of the present study was to evaluate systematically research reports on the subject in order to summarize the significant outcomes on efficacy and mechanisms.

## Methods

### Data Sources and Search Strategies

Animal experimental studies of AS-IV for DN were identified using a computerized literature search of Chinese Science and Technology Journal Database, WanFang, China National Knowledge Infrastructure, Chinese Biomedical Database, EMBASE, Cochrane library, PubMed, and Web of Science. All search strategies were performed from inception to June 2020. The following search terms were used in PubMed and were modified to suit other databases: “Astragaloside IV OR AS-IV OR Astragalus membranaceus” AND “Diabetes OR diabetic nephropathy”. The reference lists of all the eligible studies were searched carefully to obtain additional studies.

### Eligibility Criteria

The abstracts and titles of studies were screened and the full-text articles were subsequently reviewed for inclusion and exclusion by two authors (Hong Wang and Zhuang Zhuang) independently. Inclusion criteria were as follows (1) AS-IV (as monotherapy in any dose) to treat animal models of DN established in various ways; (2) nonfunctional and equal volumes of liquid (normal saline) or no treatment adopted for the control group; and (3) primary outcome measures were renal pathology, creatinine clearance rate (CCr), 24-h urinary albumin or microalbumin, 24-h urinary neutrophil gelatinase-associated lipocalin (NGAL) or N-acetyl-*β*-D-glucosaminidase (NAG), serum creatinine (SCr), blood urea nitrogen (BUN), HbA1c, or indicators of adverse reactions. The renoprotective mechanisms of AS-IV for DN were selected as secondary outcome measures. Exclusion criteria were as follows: (1) not *in vivo* studies (*in vitro* studies, clinical trials, review articles, case reports, comments, editorials, and abstracts); (2) treatment with AS-IV-based prescriptions or combinations with other drugs; (3) comparisons with other drugs with unclear efficacy; (4) no predetermined outcome index or available data; (5) not a DN model; (6) duplicate publication; and (7) no control group.

### Data Extraction

Two authors (HW and Y-YH) independently evaluated all the eligible articles for data extraction in terms of: (1) the publication year of the study and the name of the first author; (2) details regarding the animals; (3) the methods to establish animal models, the criteria for modeling successfully and the use of anesthetics in the course of experiment; (4) the therapeutic regimen and the control group; and (5) primary, secondary outcomes, and intergroup differences. If the outcomes were displayed through gradient doses of drug therapy or multiple time points, only the data of the highest dose group and peak time point group were included. The authors were contacted for specific data when the results were only rendered graphically. If a response was not received, the graph data were measured using Photoshop.

### Risk of Bias in Individual Studies

For each included study, quality assessment was carried out by two authors (ZZ and Y-YH) independently using the CAMARADES 10-item quality checklist (Macleod et al., 2004) with minor modifications (modified sections: D: blinded induction of model [group randomly after modeling, or transgenic mice, or knockout mice]; F: use of anesthetic without significant renal protective activity or nephrotoxicity). Disagreements in selecting studies, extracting data, or assessing the quality of studies were resolved by consensus or arbitration by the correspondence authors (QZ and Y-LW).

### Statistical Analysis

RevMan 5.3 software downloaded on the website (https://www.cochrane.org/) was used for data analysis where possible. When meta-analysis failed to run, comparisons between groups were performed for individual studies. The combined overall effect sizes of outcome measures were estimated by utilizing standard mean difference (SMD) with 95% confidence interval (CI). Heterogeneity was determined using the Cochrane Q-statistic test and the I2-statistic test (random effects model [I2 greater than 50%] or a fixed effects model [I2 less than or equal to 50%]). Sensitivity analyses were conducted when individual results deviated substantially. Bar graphs were drawn using Prism 6. When the probability value was less than 0.05, the difference was considered statistically significant.

## Results

### Study Selection

**Figure 2:**
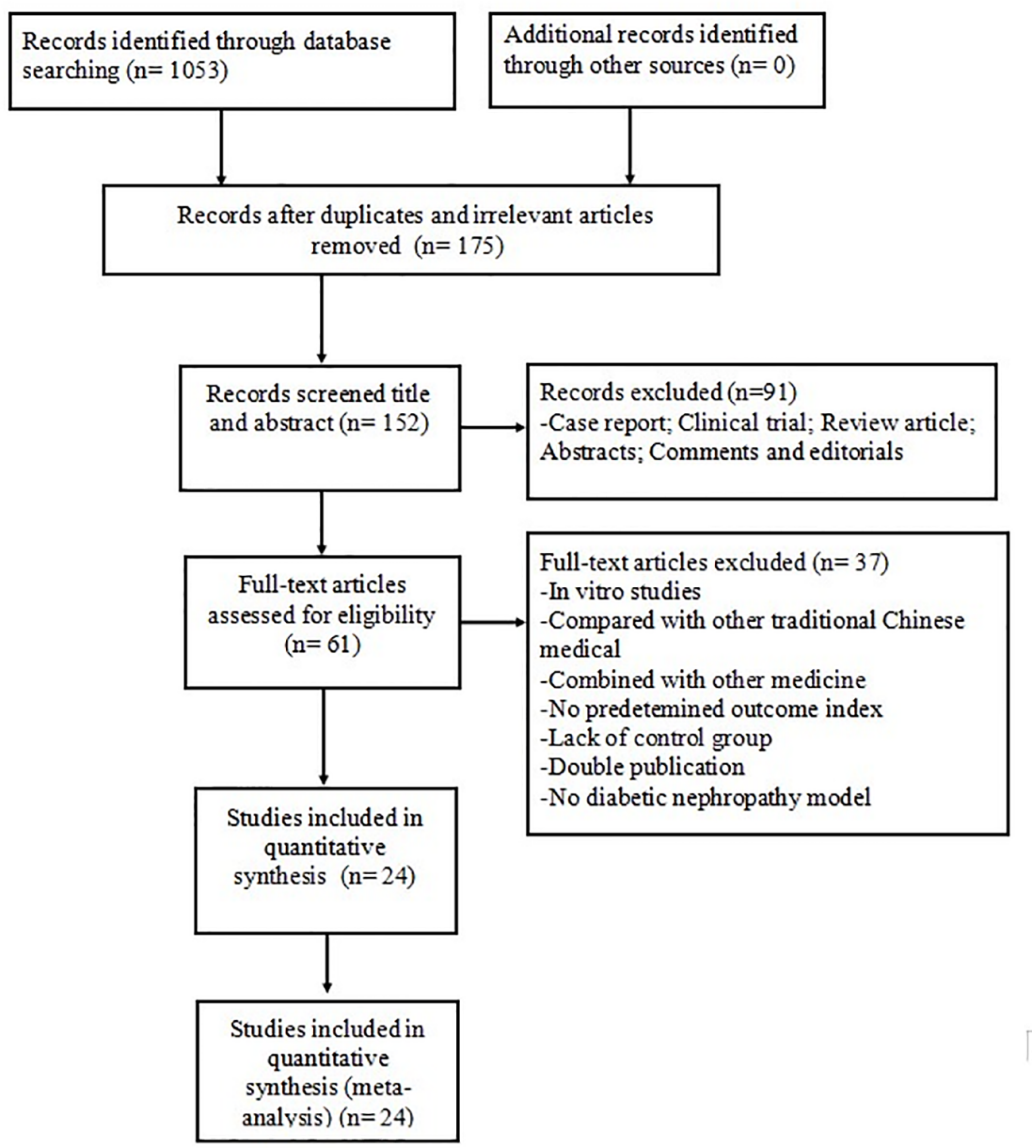
Summary of the process for identifying candidate studies.

The search strategy yielded 1,053 potentially relevant studies from the eight databases, of which 878 were duplicated or irrelevant studies. We excluded 91 non-animal studies after checking the titles and abstracts. Detailed inspection was performed for remaining 61 full-text studies; 37 of these were not considered because they presented at least one of the excluding criteria. Finally, 24 eligible studies were identified: 13 in English (Gui et al., 2012; Chen et al., 2014; Lu et al., 2015; Wang et al., 2015; Guo et al., 2016; Sun et al., 2016; Guo et al., 2017; Liu et al., 2017; Du et al., 2018; Lei et al., 2018a; Lei et al., 2018b; Fan et al., 2019; Ju et al., 2019) and 11 in Chinese (Chen and Chen, 2016; Huang et al., 2016; Li and Zhang, 2016; Ran and Ma, 2016; Wang et al., 2017; Han et al., 2018; He et al., 2018; Li, 2018; Liu et al., 2019; Ma et al., 2019; Song et al., 2020) (**Figure 2**).

### Characteristics of Included Studies

Twenty-four studies with 424 animals were included. The sample size ranged from 12 to 28 animals in each study. Sprague Dawley (SD) male rats were used in 16 studies; male Wistar rats in two studies, male C57BL/6 mice in two studies, male db/db mice in three studies, and male KKAy mice in one study. The weight of SD or Wistar rats varied between 170 and 260 g, and the weight of mice varied between 16 and 24 g. Eighteen studies established the DN model by intraperitoneal injection of streptozotocin (STZ); two studies used feeding high-fat diet for several weeks and intraperitoneal injection of STZ; four studies used mutant or transgenic mice with spontaneous diabetes (four studies used db/db mice and one study used KKAy mice) that exhibits clinical and histological features of DN resembling those found in human DN (Sharma et al., 2003; Tesch and Lim, 2010). To induce anesthesia, anesthetics were unreported in 11 studies, pentobarbital sodium was reported in seven studies, chloral hydrate was used in four studies, urethane was used in one study, and ether was used in one study. Detailed information of AS-IV in each study is displayed in **Table 1**. Twenty-two studies implemented a dose gradient of AS-IV ranging from 3 to 1.08 g•kg^−1^•d^−1^ using oral or intragastric administration. Rats received 5 mmol•d^−1^ AS-IV by oral gavage in one study and normal standard diet supplemented with AS-IV (AS-IV: feed = 5 g:1 kg) was administered to rats by oral gavage in one study. In terms of outcome measures, renal pathology was utilized as primary outcome measure in 19 studies, BUN in 13 studies, SCr in 13 studies, 24-h urinary protein in 12 studies, 24-h urinary microalbumin in six studies, 24-h urinary NAG in three studies, 24-h urinary NGAL in two studies, blood glucose (BG) in 17 studies, glycated hemoglobin (HbA1c) in four studies; CCr in one study; alanine aminotransferase (ALT) in three studies, and aspartate aminotransferase (AST) in two studies. Secondary outcomes were as follows: superoxide dismutase (SOD) was reported in three studies; catalase (CAT) in two studies; malondialdehyde (MDA) in four studies; glutathione peroxidase (GSH-PX) in three studies; tumor necrosis factor-α (TNF-α) in one study; monocyte chemotactic protein-1 (MCP-1) in one study; caspase-3 in two studies; caspase-12 in one study; Bax and Bcl-2 in two studies; transforming growth factor-*β*1 (TGF-*β*1) in five studies; Smad2/3 in two studies; Smad7 in two studies; phosphothreonine kinase (p-Akt) in four studies; phosphorylated phosphatidylinositol-3-kinase (p-PI3K) in two studies; PKR-like eukaryotic initiation factor 2A kinase 1/2 (pERK-1/2) in three studies; PTEN-induced putative kinase (PINK) in one study; Jun N-terminal kinases (JUN) in two studies; forkhead box O1 (Fox O1) in one study; mammalian target of rapamycin (mTOR) in two studies; nuclear factor kappa B (NF-*κ*B) in 2 studies; glucose-regulated protein 78 (GRP78) in three studies; sarco-endoplasmic reticulum calcium adenosine triphosphatase (SERCA) in two studies. The detailed characteristics of the included studies are displayed in **Table 2**.

**Table 1:** Information of AS-IV of each study.

Study (years)	Specifications	Source	Purity (%)	Quality control reported
Gui et al., 2012	dry powder	Xi'an Sobeo Pharmaceutical Technology Company, Limited	(≥98%)	Y-HPLC
Chen et al., 2014	dry powder	Xi'an Sobeo Pharmaceutical Technology Company, Limited	(≥98%)	Y-HPLC
Lu et al., 2015	dry powder	Chengdu Jintaihe Pharmaceutical Chemical Technology Company, Limited	(98%)	Y-HPLC
Wang et al., 2015	dry powder	Unknown	Unknown	Unknown
Chen and Chen, 2016	dry powder	Shanghai Rongbai Biotechnology Company, Limited	(≥98%)	Batch number 14051502
Guo et al., 2016	dry powder	Shanghai Bogoo Biotechnology Company, Limited	(98%)	Unknown
Huang et al., 2016	dry powder	Shanghai Pharmaceutical Development Company, Limited	Unknown	Y-HPLC
Li and Zhang, 2016	dry powder	Unknown	Unknown	Unknown
Ran and Ma, 2016	dry powder	Dalian Meilun Biotechnology Company, Limited	(98%)	BR, batch number MB1955
Sun et al., 2016	dry powder	ChengDu ConBon Biotech Company, Limited	Unknown	Y-HPLC
Guo et al., 2017	dry powder	Shanghai Bogoo Biotechnology company, Limited	(98%)	Unknown
Liu et al., 2017	dry powder	ChengDu ConBon Biotech Company, Limited	Unknown	Y-HPLC
Wang et al., 2017	dry powder	American Sigma Company	(98%)	Y-TLC
Du et al., 2018	dry powder	ChengDu ConBon Biotech Company, Limited	(≥98%)	Y-HPLC
Han et al., 2018	dry powder	ChengDu Kangbang Biotechnology Company, Limited	(≥98%)	Y-HPLC
He et al., 2018	dry powder	American Sigma Company	(98%)	Y-TLC
Li, 2018	dry powder	Nanjing Zelang Pharmaceutical Technology Company, Limited	(≥98%)	Y-HPLC, batch number ZL160305
Lei et al., 2018a	dry powder	Solarbio life sciences &technology Company, Limited	(≥98%)	Y-HPLC
Lei et al., 2018b	dry powder	Solarbio life sciences &technology Company, Limited	(≥98%)	Y-HPLC
Fan et al., 2019	dry powder	Dalian Meilun Biotechnology Company, Limited	(≥98%)	Unknown
Ju et al., 2019	dry powder	Nanjing Zelang Pharmaceutical Technology Company, Limited	(≥98%)	Y-HPLC
Liu et al., 2019	dry powder	ChengDu Ruifensi Biotechnology Company, Limited	(≥98%)	Batch number 170768
Ma et al., 2019	dry powder	Nanjing Zelang Pharmaceutical Technology Company, Limited	(≥98%)	Y-HPLC, batch number CY170925
Song 2020	dry powder	Xi'an Sobeo Pharmaceutical Technology Company, Limited	(≥98%)	Y-HPLC

HPLC, High performance liquid chromatography; TLC, thin Layer Chromatography.

**Table 2:** Characteristics of the 24 included studies.

(years)	Species (Sex, n = experimental/control group)	Weight	Model (method)	The Criteria for modeling successfully	Anesthetic	Treatment group (Method to astragal sides)	Control group	Outcome index (time)	Intergroup differences
Gui et al., 2012	SD rats (male, 8/8)	180–200 g	By intraperitoneal injection of STZ (65 mg/kg)	Rats with a blood glucose level over 300 mg/dl in 3 different times after 72 h of STZ injection	Pentobarbital sodium	By oral gavage of AS-IV (10 mg/kg, qd) in 2 weeks before STZ injection and lasted 14 weeks	By oral gavage of an equal volume of CMC in 2 weeks before STZ injection and lasted 14 weeks	1. Renal pathology 2. BUN and SCr 3. HbA1c 4. BG 5. ALT 6. SOD and MDA 7. CAT 8. Caspase-3 9. Bax and Bcl-2	1. P < 0.05 2. P > 0.05 3. P < 0.05 4. P < 0.05 5. P < 0.05 6. P < 0.05 7. P < 0.05 8. P < 0.05 9. P < 0.05
Chen et al., 2014	SD rats (male, 8/8)	180–200 g	By intraperitoneal injection of STZ (65 mg/kg)	Rats with a blood glucose level over 300 mg/dl in 3 different times after 72 h of STZ injection	Pentobarbital sodium	By oral gavage of AS-IV (10 mg/kg, qd) in 2 weeks before STZ injection and lasted 14 weeks	By oral gavage of an equal volume of CMC in 2 weeks before STZ injection and lasted 14 weeks	1. Renal pathology 2. 24-h urinary protein 3. BUN and SCr 4. BG 5. ALT and AST 6. Integrin *α*3, *β*1 subunits and ILK	1. P < 0.05 2. P < 0.05 3. P < 0.05 4. P < 0.05 5. P < 0.05 6. P < 0.05
Lu et al., 2015	SD rats (male, 14/14)	180–220 g	By intraperitoneal injection of STZ (125 mg/kg, qd) for 2 consecutive days	Rats with a blood glucose level over 16.7 mmol/L in 3 different times after 72 h of STZ injection	NM	By oral gavage of AS-IV (40 mg/kg, qd) after STZ injection and lasted 8 weeks	By oral gavage of an equal volume of NS after STZ injection and lasted 8 weeks	1. Renal pathology 2. 24-h urinary microalbumin 3. BG 4. integrin *α*4 and *β*1 subunits and ILK	1. P < 0.05 2. P < 0.05 3. P < 0.05 4. P < 0.05
Wang et al., 2015	SD rats (male, 14/14)	180–200 g	By intraperitoneal injection of STZ (50 mg/kg, qd) for 5 consecutive days	Rats with a blood glucose level over 300 mg/dl in 3 different times after 72 h of STZ injection	Pentobarbital sodium	By oral gavage of AS-IV (10 mg/kg, qd) in 2 weeks after STZ injection and lasted 8 weeks	By oral gavage of an equal volume of NS in 2 weeks after STZ injection and lasted 8 weeks	1. Renal pathology 2. 24-h urinary protein 3. BUN and SCr 4. Kidney/body weight ratio 5. BG 6. Total and phosphorylated PERK, eIF2a, and JNK 7. GRP78 and ORP150	1. P < 0.05 2. P < 0.05 3. P < 0.05 4. P < 0.05 5. P < 0.05 6. P < 0.05 7. P < 0.05
Chen and Chen, 2016	SD rats (male, 10/10)	180–200 g	By intraperitoneal injection of STZ (40 mg/kg)	Rats with a blood glucose level over 16.6 mmol/L in 3 different times after 72 h of STZ injection	Chloral hydrate (35 mg/kg)	By oral gavage of AS-IV (1 mmol/L, 5 ml, qd) after STZ injection and lasted 30 days	By oral gavage of an equal volume of NS in after STZ injection and 30 days	1. Renal pathology 2. 24-h urinary protein 3. UCr 4. BG	1. P < 0.05 2. P < 0.05 3. P < 0.05 4. P < 0.05
Guo et al., 2016	db/db mice (male, 10/10)	NM	BKS.cg-m +/+ Leprdb/J mice (Spontaneous diabetes )	NM	NM	By oral gavage of AS-IV (18 mg/kg, qd) at 8 weeks of age and lasted 8 weeks	By oral gavage of an equal volume of CMC at 8 weeks of age and lasted 8 weeks	1. Renal pathology 2. BUN and SCr 3. Kidney/body weight ratio 4. HbA1c 5. BG 6. HOMA-IR 7. TNF-α 8. MCP-1 9. Serum insulin 10. SERCA	1. P < 0.05 2. P < 0.05 3. P < 0.05 4. P < 0.05 5. P < 0.05 6. P < 0.05 7. P < 0.05 8. P < 0.05 9. P < 0.05 10. P < 0.05
Huang et al., 2016	Wistar rats (male, 10/10)	170–230 g	By intraperitoneal injection of STZ (60 mg/kg)	Rats with a blood glucose level over 16.7 mmol/L and 24-h urinary protein level over 30mg/kg/d after 72 h of STZ injection	Urethane (100mg/kg)	By oral gavage of AS-IV (3 mg/kg, qd) after STZ injection and lasted 8 weeks	By oral gavage of an equal volume of citrate buffer after STZ injection and lasted 8 weeks	1. 24-h urinary protein 2. BUN and SCr 3. BG 4. SOD, MDA, and GSH-Px 5. TGF-*β*1 mRNA 6. CAT mRNA	1.P<0.05 2.P<0.05 3. P < 0.05 4. P < 0.05 5. P < 0.05 6. P < 0.05
Li and Zhang, 2016	SD rats (male, 10/10)	NM	By intraperitoneal injection of STZ (60 mg/kg)	Rats with a blood glucose level over 13.9 mg/kg after 1 week of STZ injection	Pentobarbital sodium	By oral gavage of AS-IV (1.08 g/kg, qd) after STZ injection and lasted 6 weeks	By oral gavage of an equal volume of NS after STZ injection and lasted 6 weeks	1. Renal pathology 2. 24-h urinary microalbumin 3. Kidney/body weight ratio 4. BG 5. TGF-*β*1 6. Collagen I, II, III 7. *α*-SMA 8. T*β*RI, T*β*RII 9. Smad 2/3 and Smad 4	1. P < 0.05 2. P < 0.05 3. P < 0.05 4. P < 0.05 5. P < 0.05 6. P < 0.05 7. P < 0.05 8. P < 0.05 9. P < 0.05
Ran and Ma, 2016	SD rats (male, 10/10)	240–260 g	By intraperitoneal injection of STZ (65 mg/kg)	Rats with a blood glucose level over 16.7 mmol/L after 72 h of STZ injection	NM	By oral gavage of AS-IV (5 mg/kg, qd) after STZ injection and lasted 8 weeks	By oral gavage of an equal volume of distilled water after STZ injection and lasted 8 weeks	1. Histological structure of kidney under transmission electron microscopy 2. 24-h urinary protein 3. 24-h urinary microalbumin 4. BG 5. Adiponectin	1. P < 0.05 2. P < 0.05 3. P < 0.05 4. P < 0.05 5. P < 0.05
Sun et al., 2016	db/db mice (male, 8/8)	NM	BKS.Cg-Dock7^m^ +/+Lepr^db^/JNju) mice (Spontaneous diabetes )	NM	NM	By oral gavage of AS-IV (1 g/kg, qd) at 8 weeks of age and lasted 12 weeks	By oral gavage of an equal volume of NS at 8 weeks of age and lasted 12 weeks	1. Renal pathology 2. 24-h urinary protein 3. 24-h urinary NAG and NGAL 4.HbA1c 5.Serum insulin 6.ALT and AST 7.Urinary TGF-*β*1 8.p-Akt 9.β-actin 10.p-mTOR 11. p-NF-*κ*B 12. p-ERK1/2 13. BG	1. P < 0.05 2. P < 0.05 3. P < 0.05 4. P < 0.05 5. P < 0.05 6. P < 0.05 7. P < 0.05 8. P < 0.05 9. P < 0.05 10. P < 0.05 11 .P < 0.05 12. P < 0.05 13. P < 0.05
Guo et al., 2017	C57BL/6J mice (male, 12/12)	NM	By intraperitoneal injection of STZ (100 mg/kg, qd) for 2 consecutive days	Mice with a blood glucose level over 350 mg/dl after 1 week of STZ injection	NM	By oral gavage of AS-IV (12 mg/kg, qd) after STZ injection and lasted 8 weeks	By oral gavage of an equal volume of NS after STZ injection and lasted 8 weeks	1. Renal pathology 2. BUN and SCr 3. GRP78 4. TF6 5. p-ERK1/2 6. p-eIF2*α* and p-IRE1*α* 7. CHOP 8. TRAF2 9. p-JNK 10. Caspase-12 11. SERCA	1. P < 0.05 2. P < 0.05 3. P < 0.05 4. P < 0.05 5. P < 0.05 6. P < 0.05 7. P < 0.05 8. P < 0.05 9. P < 0.05 10. P < 0.05 11. P < 0.05
Liu et al., 2017	db/db mice (male, 6/6)	NM	C57BL/KSJ mice (Spontaneous diabetes)	NM	Pentobarbital sodium (75 mg/kg)	By oral gavage of AS-IV (1 g/kg, qd) at 8 weeks of age and lasted 12 weeks	By oral gavage of an equal volume of NS at 8 weeks of age and lasted 12 weeks	1. Renal pathology 2. 24-h urinary protein 3. 24-h urinary NAG 4. Drp-1, Fis-1, and MFF 5. Mfn-1, Mfn-2,and OPA-1 6. PINK1, Parkin, p-Parkin (Ser 65), and LC-3II	1. P < 0.05 2. P < 0.05 3. P < 0.05 4. P < 0.05 5. P < 0.05 6. P < 0.05
Wang et al., 2017	KKAy mice (male, 10/10)	22–24 g	By feeding with high-fat diet for 14 consecutive weeks before experiment and lasted 14 weeks	Mice with a blood glucose level over 13.9 mmol/L after 14 weeks of high-fat diet	Aether	By oral gavage of AS-IV (40 mg/kg, qd) after 14 weeks of high-fat diet and lasted 10 weeks	By oral gavage of an equal volume of NS after 14 weeks of high-fat diet and lasted 10 weeks	1. Renal pathology 2. BG 3. *α*-SMA 4. TGF-*β*1 5. Smad2/3	1. P < 0.05 2. P < 0.05 3. P < 0.05 4. P < 0.05 5. P < 0.05
Du et al., 2018	Wistar rats (male, 8/8)	180–220 g	By intraperitoneal injection of STZ (60 mg/kg)	Rats with a blood glucose level over 13.8 mg/kg after 72 h of STZ injection	NM	By oral gavage of AS-IV (16 mg/kg, qd) after STZ injection and lasted 8 weeks	By oral gavage of an equal volume of NS after STZ injection and lasted 8 weeks	1. BUN and SCr 2. UCr 3. *β*2-MG 4. MDA, CAT and GSH-Px 5. T-AOC	1. P< 0.05 2. P < 0.05 3. P < 0.05 4. P < 0.05 5. P < 0.05
Han et al., 2018	C57BL/6J (male, 6/6)	NM	By intraperitoneal injection of STZ (200 mg/kg)	Mice with a blood glucose level over 16.7 mmol/L	NM	By oral of feed supplemented with AS-IV (AS-IV:feed = 5g:1kg) and lasted 8 weeks	By oral of an equal quality of standard feed and lasted 8 weeks	1.Histological structure of kidney under transmission electron microscopy 2. Renal pathology 3. 24-h urinary protein 4. 24-h urinary NAG and NGAL 5. Kidney/body weight ratio 6. p-Akt 7. p-mTOR	1. P < 0.05 2. P < 0.05 3. P < 0.05 4. P < 0.05 5. P < 0.05 6. P < 0.05 7. P < 0.05
He et al., 2018	SD rats (male, 10/10)	180–220 g	By intraperitoneal injection of STZ (65 mg/kg)	Rats with a blood glucose level over 16.7 mmol/L after 72 h of STZ injection	NM	By oral gavage of AS-IV (10 mg/kg, qd) after STZ injection and lasted 8 weeks	By oral gavage of an equal volume of CMC after STZ injection and lasted 8 weeks	1. Renal pathology 2. 24-h urinary protein 3. p-130Cas	1. P < 0.05 2. P < 0.05 3. P < 0.05
Li, 2018	SD rats (male, 11/10)	180–220 g	By intraperitoneal injection of STZ (55 mg/kg)	Rats with a blood glucose level over 16.7 mmol/L after 72 h of STZ injection	Chloral hydrate (35 mg/kg)	By oral gavage of AS-IV (80 mg/kg, qd) after STZ injection and lasted 8 weeks	By oral gavage of an equal volume of CMC after STZ injection and lasted 8 weeks	1. Renal pathology 2. 24-h urinary microalbumin 3. Kidney/body weight ratio 4. BUN and SCr 5. BG 6. MDA, SOD, and GSH-Px 7. Nrf2, NQO1, and HO1 8. TGF-*β*1	1. P < 0.05 2. P < 0.05 3. P < 0.05 4. P < 0.05 5. P < 0.05 6. P < 0.05 7. P < 0.05 8. P < 0.05
Lei et al., 2018a	SD rats (male, 6/6)	200g	By intraperitoneal injection of STZ (65 mg/kg)	Rats with a blood glucose level over 300 mg/dl after 48 h of STZ injection	NM	By oral gavage of AS-IV (5 mg/kg, qd) after STZ injection and lasted 12 weeks	By oral gavage of an equal volume of citrate buffer after STZ injection and lasted 12 weeks	1. 24-h urinary protein 2. TUG1 level 3. The TRAF5 mRNA and protein level	1. P < 0.05 2. P < 0.05 3. P < 0.05
Lei et al., 2018b	SD rats (male, 6/6)	200–220 g	By intraperitoneal injection of STZ (65 mg/kg)	Rats with a blood glucose level over 300 mg/dl after 48 h of STZ injection	Pentobarbital sodium	By oral gavage of AS-IV (5 mg/kg, qd) after STZ injection and lasted 12 weeks	By oral gavage of an equal volume of citrate buffer after STZ injection and lasted 12 weeks	1. BUN and SCr 2. TRAF5 3. miR-378	1. P < 0.05 2. P < 0.05 3. P < 0.05
Fan et al., 2019	SD rats (male, 6/6)	180-200 g	By intraperitoneal injection of STZ (65 mg/kg)	Rats with a blood glucose level over 300 mg/dl after 48 h of STZ injection	pentobarbital sodium	By oral gavage of AS-IV (10 mg/kg, qd) after STZ injection and lasted 8 weeks	By oral gavage of an equal volume of CMC after STZ injection and lasted 8 weeks	1. Renal pathology 2. HbA1c 3. BUN and SCr 4. Nitric oxide 5. Endothelial nitric oxide synthase 6. Body weight 7. BG 8. 24-h urinary protein 9. Cell apoptosis rate	1. P < 0.05 2. P < 0.05 3. P < 0.05 4. P < 0.05 5. P < 0.05 6. P < 0.05 7. P < 0.05 8. P < 0.05 9. P < 0.05
Ju et al., 2019	SD rats (male, 8/8)	180–220 g	By feeding with high-fat diet for 6 consecutive weeks before modeling+ by intraperitoneal injection of STZ (35 mg/kg)	Rats with a blood glucose level over 16.7 mmol/L after 72 h of STZ injection	Chloral hydrate	By oral gavage of AS-IV (80 mg/kg, qd) in 1 week after STZ injection and lasted 8 weeks	By oral gavage of an equal volume of CMC in 1 week after STZ injection and lasted 8 weeks	1. Renal pathology 2. 24-h urinary microalbumin 3. BUN and SCr 4. Kidney/body weight ratio 5. Ratio of urine total protein to UCr 6. CCr 7. BG 8. TG and TC 9. Bax, Bcl-2, and Bax/Bcl-2 10. Caspase-3 11. β-actin 12. GRP78	1. P < 0.05 2. P < 0.05 3. P < 0.05 4. P < 0.05 5. P < 0.05 6. P < 0.05 7. P < 0.05 8. P < 0.05 9. P < 0.05 10. P < 0.05 11. P < 0.05 12. P < 0.05
Liu et al., 2019	SD rats (male, 10/10)	180-200 g	By intraperitoneal injection of STZ (50 mg/kg)	Rats with a blood glucose level over 16.7 mmol/L after 72 h of STZ injection	NM	By oral gavage of AS-IV (60 mg/kg, qd) after STZ injection and lasted 8 weeks	By oral gavage of an equal volume of distilled water after STZ injection and lasted 8 weeks	1. BG 2. Urine protein 3. BUN and SCr 4. p-PI3K 5. p-Akt 6. NF-κB 7. Renal hypertrophy index	1. P < 0.05 2. P < 0.05 3. P < 0.05 4. P < 0.05 5. P < 0.05 6. P < 0.05 7. P < 0.05
Ma et al., 2019	SD rats (male, 13/10)	180–220 g	By feeding with high-fat and high-suga diet for 6 consecutive weeks before modeling+ by intraperitoneal injection of STZ (35 mg/kg)	Rats with a blood glucose level over 16.7 mmol/L after 72 h of STZ injection	Chloral hydrate	By oral gavage of AS-IV (80 mg/kg, qd) after STZ injection and lasted 8 weeks	By oral gavage of an equal volume of CMC after STZ injection and lasted 8 weeks	1. Renal pathology 2. 24-h urinary microalbumin 3. 24-h urinary protein 4. BUN and SCr 5. Kidney/body weight ratio 6. BG 7. p-Fox O1/Fox O1 8. p-PI3K/PI3K 9. p-Akt/Akt	1. P < 0.05 2. P < 0.05 3. P < 0.05 4. P < 0.05 5. P < 0.05 6. P < 0.05 7. P < 0.05 8. P < 0.05 9. P < 0.05
Song et al., 2020	SD rats (male, 8/8)	200–220 g	By intraperitoneal injection of STZ (58 mg/kg)	Rats with a blood glucose level over 16.7 mmol/L after 72 h of STZ injection	NM	By oral gavage of AS-IV (10 mg/kg, qd) after STZ injection and lasted 12 weeks	By oral gavage of an equal volume of distilled water after STZ injection and lasted 12 weeks	1. Renal pathology 2. 24-h urinar**y p**rotein 3. BUN and SCr 4. Kidney/body weight ratio 5. BG 6. Desmin	1. P < 0.05 2. P < 0.05 3. P > 0.05 4. P < 0.05 5. P < 0.05 6. P < 0.05

ALT, alanine aminotransferase; AST, aspartate aminotransferase; BG, blood glucose; BUN, blood urea nitrogen; β2-MG, β2-microglobulin; Cas, crk-associated substrate; CAT, catalase; CCr, creatinine clearance rate; CHOP, CCAAT/enhancer-binding protein homologous protein; CMC, carboxymethyl cellulos; COl-IV, type IV collagen; Cr, creatinine; Drp-1, dynamin-related protein 1; Fis-1, mitochondrial fission protein 1; Fox O1, forkhead box O1; HbA1c, glycated hemoglobin; GRP78, glucose-regulated protein 78; GSH-PX, glutathione peroxidase; HbA1c, glycated hemoglobin; HOMA-IR, homeostatic model assessment of insulin resistance; HDL, high density lipoprotein; ILK, integrin-linked kinase; IL-1β, interleukin-1β; JNK, Jun N-terminal kinases; LDL, low-density lipoprotein; LN, laminin; MCP-1, monocyte chemotactic protein-1; MDA, malondialdehyde; MFF, mitochondrial fission factor; Mfn-1, rabbit antimitofusin 1; Mfn-2, rabbit antimitofusin 2; mTOR, mammalian target of rapamycin; NAG, N-acetyl-β-D-glucosaminidase; NGAL, neutrophil gelatinase-associated lipocalin; NM, not mentioned; NQO1, nad(p)h quinnone dehydrogenase 1; Nrf2, nuclear factor E2 related-factor 2; NS, normal saline; OPA-1, mouse anti-optic atrophy 1; ORP150, 150 kDa oxygen-regulated protein; PERK, PKR-like eukaryotic initiation factor 2A kinase; p-PI3K, phosphorylated phosphatidylinositol-3-kinase; PINK, PTEN-induced putative kinase; SCr, serum creatinine; SD rats, Sprague Dawley rats; SERCA, sarco endoplasmic reticulum calcium adenosine triphosphatase; SOD, superoxide dismutase; STZ, Streptozotocin; T-AOC, total anti-oxidative capacity; TC, Serum total cholesterol; TG, Triglyceride; TP, Total protein; TNF-α, tumor necrosis factor-α; TRAF2, TNF receptor associated factor 2; UCr, urinary creatinine.

### Study Quality

The CAMARADES 10-item quality checklist was adopted to judge the risk of bias of each study and the number of criteria met varied from 3/10 to 8/10 with the average of 5.4. The review authors’ judgments about each risk of bias item for each included study are summarized in **Table 3**.

**Table 3:** Risk of bias of the included studies.

Study	A	B	C	D	E	F	G	H	I	J	Total
Gui et al., 2012	**√**	**√**				**√**			**√**	**√**	**5**
Chen et al., 2014	**√**	**√**	**√**			**√**			**√**	**√**	**6**
Lu et al., 2015	**√**		**√**							**√**	**3**
Wang et al., 2015	**√**	**√**				**√**				**√**	**4**
Chen and Chen, 2016	**√**	**√**	**√**			**√**				**√**	**5**
Guo et al., 2016	**√**	**√**	**√**	**√**					**√**	**√**	**6**
Huang et al., 2016	**√**	**√**	**√**			**√**				**√**	**5**
Li and Zhang, 2016	**√**	**√**	**√**	**√**		**√**				**√**	**6**
Ran and Ma, 2016	**√**	**√**	**√**						**√**	**√**	**5**
Sun et al., 2016	**√**	**√**	**√**	**√**					**√**	**√**	**6**
Guo et al., 2017	**√**	**√**	**√**							**√**	**4**
Liu et al., 2017	**√**	**√**	**√**	**√**		**√**			**√**	**√**	**7**
Wang et al., 2017	**√**	**√**	**√**	**√**		**√**			**√**	**√**	**7**
Du et al., 2018	**√**		**√**			**√**			**√**	**√**	**5**
Han et al., 2018	**√**	**√**	**√**	**√**						**√**	**5**
He et al., 2018	**√**	**√**	**√**							**√**	**4**
Li et al., 2018		**√**	**√**	**√**		**√**			**√**	**√**	**6**
Lei et al., 2018a	**√**	**√**		**√**					**√**	**√**	**5**
Lei et al., 2018b	**√**	**√**	**√**			**√**			**√**	**√**	**6**
Fan et al., 2019	**√**	**√**		**√**		**√**			**√**	**√**	**6**
Ju et al., 2019	**√**	**√**	**√**	**√**		**√**	**√**		**√**	**√**	**8**
Liu et al., 2019	**√**	**√**	**√**	**√**						**√**	**5**
Ma et al., 2019	**√**	**√**	**√**	**√**		**√**			**√**	**√**	**7**
Song et al., 2020	**√**	**√**	**√**							**√**	**4**

Studies fulfilling the criteria of: A: peer reviewed publication; B: control of temperature; C: random allocation to treatment or control; D: blinded induction of model (group randomly after modeling or transgenic mice or knockout mice); E: blinded assessment of outcome; F: use of anesthetic without significant renal protective activity or nephrotoxicity; G: appropriate animal model (aged, hyperlipemia or hypertensive); H: sample size calculation; I: compliance with animal welfare regulations (including three or more of the following points: preoperative anesthesia, postoperative analgesia, nutrition, disinfection, environment temperature, environment humidity, circadian rhythm, and euthanasia); J: statement of potential conflict of interests.

### Effectiveness

#### Renal Pathology

Compared with the control group, AS-IV treatment inhibited podocyte apoptosis and ameliorate podocyte foot process effacement significantly in nine studies as assessed using electron or light microscopy. Treatment alleviated mesangial cell proliferation and the broadening of mesangial matrix significantly in 14 studies, but not to a significant degree in one study. There was inhibition of basement membrane thickening in four studies, narrowing the enlarged glomerular volume and alleviated the level of glomerular fibrosis in six studies, and alleviation of wall edema in proximal tubules and reduction of apoptosis of renal tubular epithelial cells in four studies.

#### CCr, BUN, and SCr

**Figure 3:**
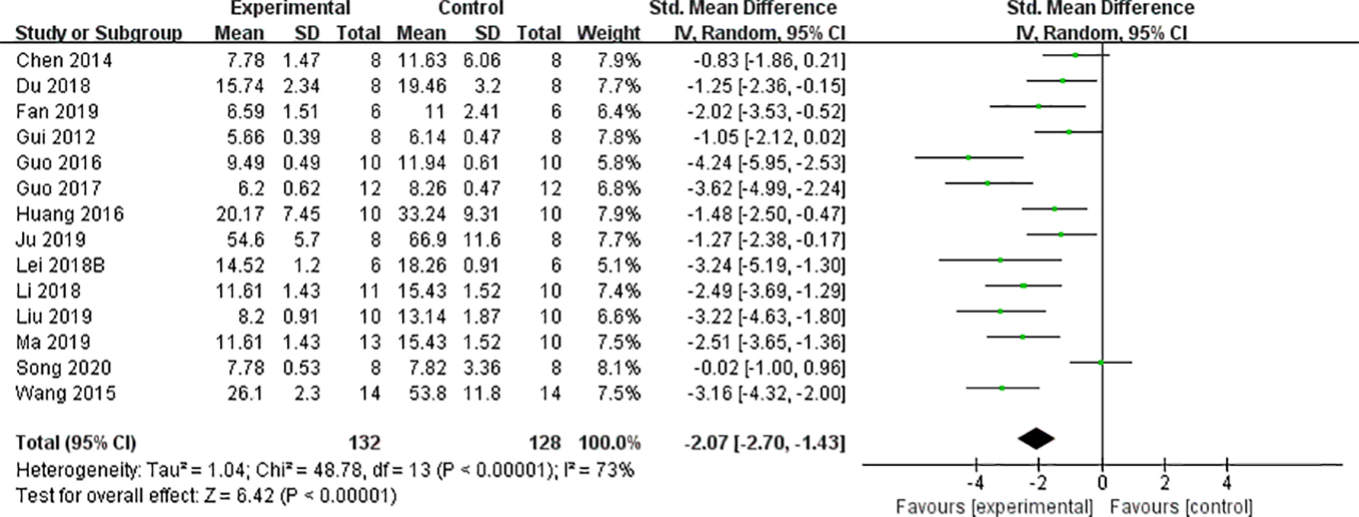
Effect of astragaloside IV on blood urea nitrogen *vs* control.

**Figure 4:**
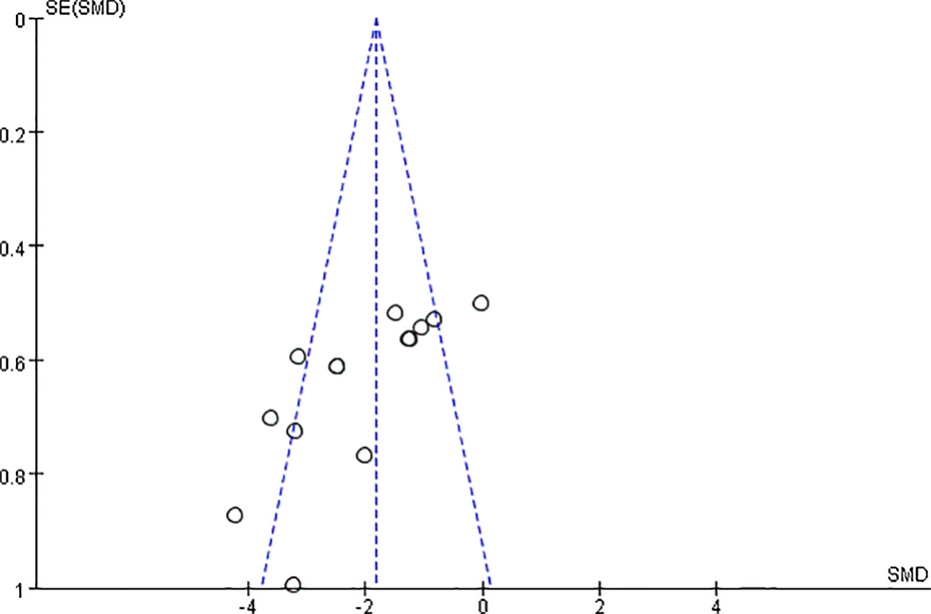
The funnel plot: effect of astragaloside IV on blood urea nitrogen.

**Figure 5:**
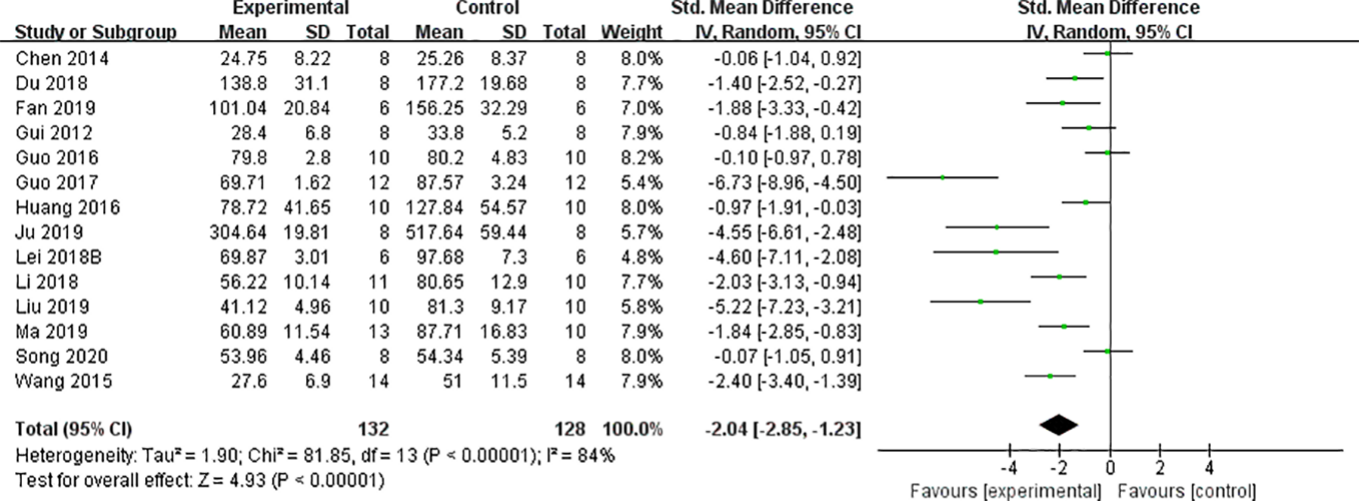
Effect of astragaloside IV on serum creatinine *vs* control.

We found that CCr was reduced significantly by AS-IV compared with the control group (P < 0.05). Meta-analysis of 13 studies indicated BUN was reduced significantly by AS-IV compared with the control group (**Figure 3**). The publication bias funnel indicated that there is no substantial publication bias (**Figure 4**). Meta-analysis of 13 studies indicated SCr was decreased significantly by AS-IV (**Figure 5**). Sensitivity analyses of indicators of BUN and SCr were carried out, and these indicated that heterogeneity did not decline significantly after eliminating any study.

#### Levels of 24-h Urinary Albumin, Microalbumin, NAG, and NGAL

**Figure 6:**
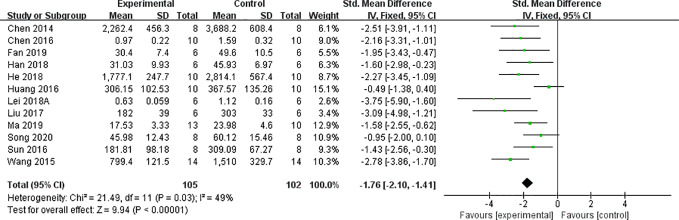
Effect of astragaloside IV on 24-h urinary albumin *vs* control.

**Figure 7:**
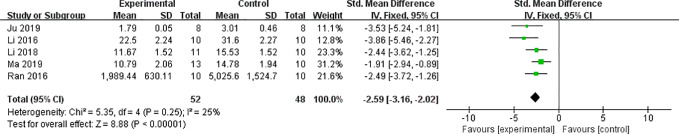
Effect of astragaloside IV on 24-h urinary microalbumin *vs* control.

**Figure 8:**
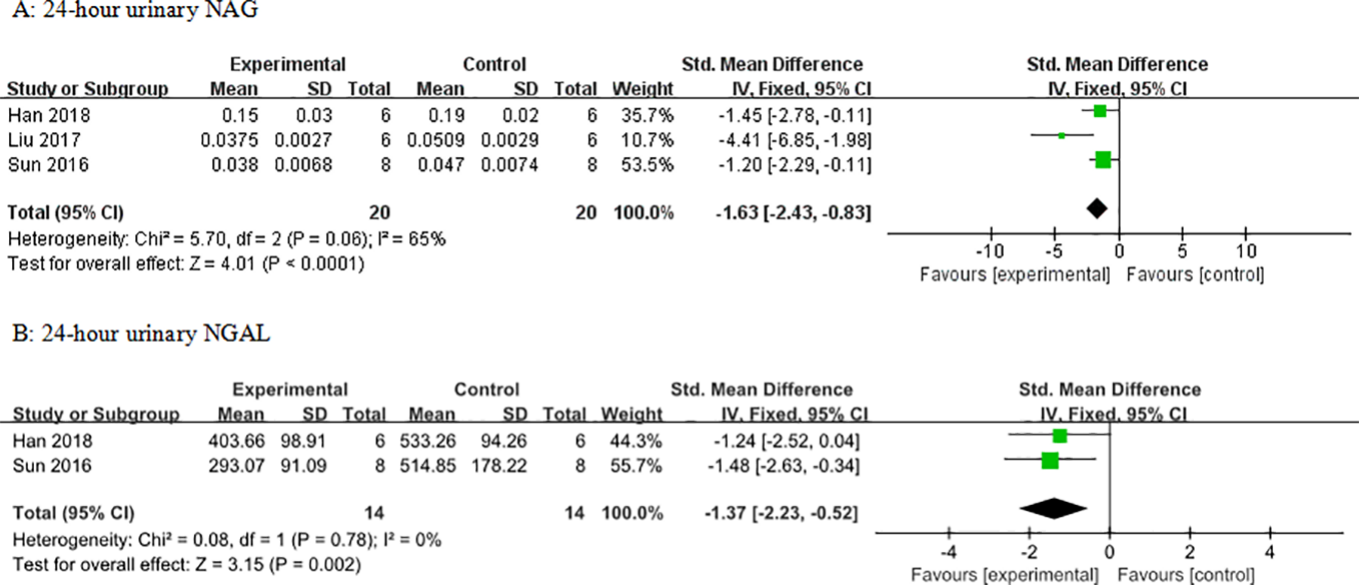
Effect of astragaloside IV on **(A)** 24-h urinary N-acetyl-*β*-D-glucosaminidase (NAG) and **(B)** neutrophil gelatinase-associated lipocalin (NGAL) *vs* control.

Meta-analysis of 12 and six studies reported that 24-h urinary albumin (**Figure 6**) and 24-h urinary microalbumin, respectively, were reduced significantly by AS-IV. After sensitivity analyses, one study was removed because the DN experimental model in that study was established using intraperitoneal injection of STZ with 125 *vs ≤65 mg/kg in other studies. Subsequently, heterogeneity of this indicator decreased substantially* (**Figure 7**). Meta-analysis of three and two studies indicated 24-h urinary NAG (**Figure 8**) and 24-h urinary NGAL (**Figure 8**), respectively, were reduced significantly by AS-IV.

#### BG and HbA1c

**Figure 9:**
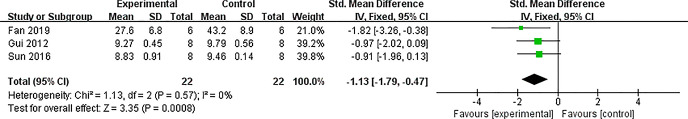
Effect of astragaloside IV on HbA1c *vs* control.

Seventeen studies used BG as an outcome measure, and all of these indicated that BG was decreased by AS-IV compared with the control group (P < 0.05). HbA1c in four studies failed to pool in the analysis because the ways of calculating HbA1c varied. Meta-analysis of the remaining three studies indicated that HbA1c level was significantly decreased by AS-IV (**Figure 9**).

#### Adverse Reactions

**Figure 10:**
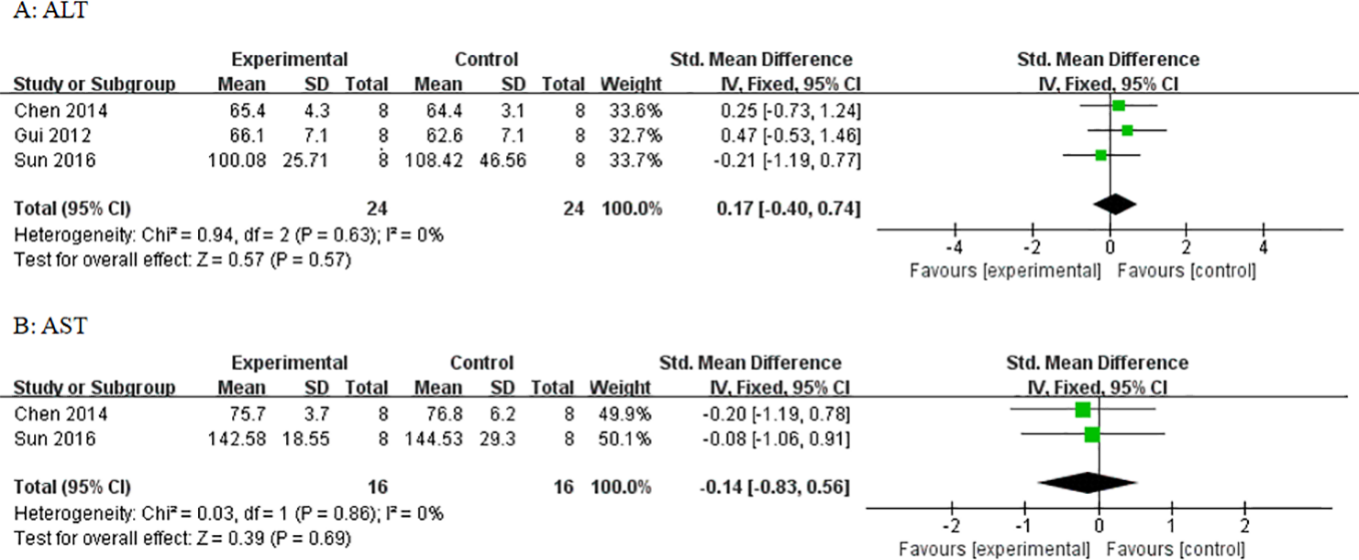
Effect of astragaloside IV on **(A)** alanine aminotransferase (ALT) and **(B)** aspartate aminotransferase (AST) *vs* control.

**Figure 11:**
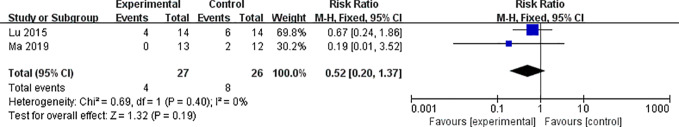
Effect of astragaloside IV on mortality of rats *vs* control.

Three studies utilized ALT and three studies utilized AST as outcome measure to assess the adverse effects of AS-IV on the liver. Meta-analysis of these studies showed no significant effect of AS-IV on ALT (**Figure 10**) or AST (**Figure 10**). Two studies reported mortality as an outcome measure, and meta-analysis of both studies showed that there was no significant difference in mortality between the AS-IV group and the control group (**Figure 11**). Two studies reported external manifestations in animals (including, activity, and glossiness of fur) with the AS-IV + STZ group showing better results than the STZ group.

#### Renoprotective Mechanisms

**Figure 12:**
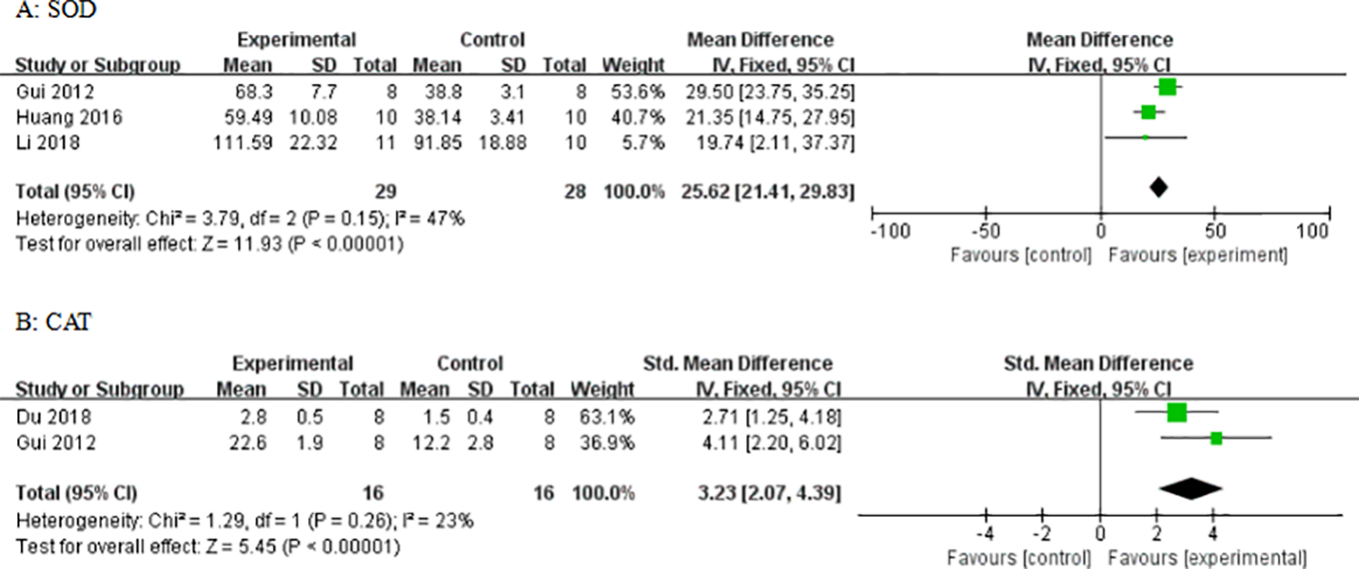
Effect of astragaloside IV on **(A)** superoxide dismutase (SOD) and **(B)** catalase (CAT) *vs* control.

Compared with control groups, meta-analysis of three studies and two studies showed that AS-IV increased SOD (**Figure 12**) and CAT (**Figure 12**), respectively. In three studies, there was increased GSH-px (P < 0.05). In four studies, there were decreased levels of MDA (P < 0.05). In one study, there were decreased levels of TNF-*α* and MCP-1. In two studies, there were decreased levels of caspase-3. In one study, there were decreased levels of caspase-12. In two studies, there were decreased levels of Bax and increased levels of Bcl-2. In five studies, there were decreased levels of TGF-*β*1. In two studies, there were decreased levels of Smad2/3. In two studies, there were increased levels of Smad7. In two studies, there were decreased levels of NF-*κ*B. In three studies, there were decreased levels of GRP78.

### Subgroup Analysis

**Figure 13:**
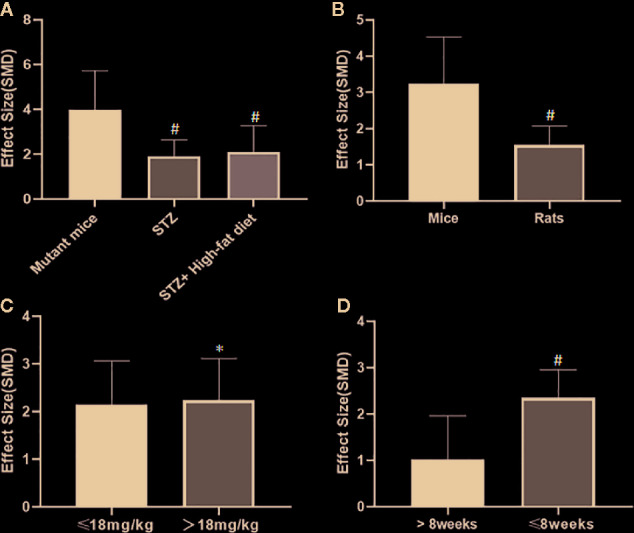
Effect of astragaloside IV on blood urea nitrogen in subgroups. **(A)** Induction type; **(B)** Species; **(C)** AS-IV dose; **(D)** Duration of treatment. ^#^P < 0.05 *vs* control; ^*^P > 0.05 *vs* control.

**Figure 14:**
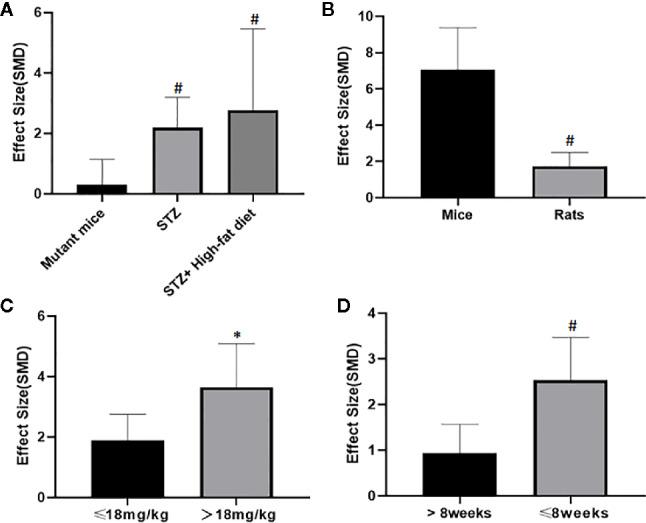
Effect of astragaloside IV on serum creatinine in subgroups. **(A)** Induction type; **(B)** Species; **(C)** AS-IV dose; **(D)** Duration of treatment. ^#^P < 0.05 *vs* control; ^*^ P > 0.05 *vs* control.

Potential confounding factors (including various methods of modeling, different animal species, varying doses of AS-IV and duration of treatment) that may have increased the heterogeneity of outcome measure were explored using stratified analysis of the BUN and SCr. The subgroup analysis of Scr showed basically the same result as that of BUN (**Figures 13** and **14**). In the subgroup analysis of the various modeling methods, the effect size of the model induced by mutant mice showed a different result than that of the models induced by administration of STZ; the method of low dose STZ injection combined with high-fat diet (SMD −4.24 *vs.* SMD −1.89 *vs.* SMD −1.42 P < 0.05, **Figure 13**; SMD −0.1 *vs.* SMD −2.09 *vs.* SMD −3.04 P < 0.05, **Figure 14**), and the heterogeneity of the three groups decreased substantially. The group in which DN was induced in C57BL/6J mice showed greater effect size than in rats (SMD −3.62 *vs.* SMD −1.64, P < 0.05, **Figure 13**; SMD −6.73 *vs.* SMD −1.88, P < 0.05, **Figure 14**), and the heterogeneity of two groups decreased substantially. No difference was seen between the high-dose AS-IV group (>18 mg/kg, QD) and the low-dose group (≤18 mg/kg, QD) (SMD −1.96 *vs.* SMD −2.04, P > 0.05, **Figure 13**; SMD −3.17 *vs.* SMD −1.58, P > 0.05, **Figure 14**). Notably, the longer period of AS-IV treatment showed a smaller effect size than the shorter treatment lasting 8 weeks or less with a small drop in the heterogeneity of the two groups (SMD −1.23 *vs.* SMD −2.26, P < 0.05, **Figure 13**; SMD −0.92 *vs.* SMD −2.04, P < 0.05, **Figure 14**).

## Discussion

### Summary of Evidence

This first-ever preclinical systematic review including 24 studies with 424 animals measured the efficacy of AS-IV for treatment of DN. The quality of included original studies was moderate. Our findings suggest that AS-IV is a multifaceted renoprotective candidate drug for treatment of DN.

### Limitations

There are some limitations of this meta-analysis and systematic review: (1) though blinding induction of model was reported in 10 studies, most of the studies had flaws with respect to blinding assessment of outcome and sample size calculation (Moher et al., 2015); (2) selection bias was unavoidable because only eight frequently-used databases were searched for English and Chinese language articles; (3) the absence of negative studies might have led to the true effect of AS-IV being overestimated (Franco et al., 2014); (4) though animal welfare regulations were observed in 14 studies, no study reported disinfection when the experimenter performed intrusive procedures such as intraperitoneal injection, subcutaneous injection, and blood glucose measurement, all of which are regarded as crucial steps in animal models, especially in diabetic models; and (5) no study utilized animals with relevant comorbidities.

### Implications

It is commendable that the blinding protocols were reported in ten studies; nevertheless, the average score of the included studies was moderate. According to the ARRIVE guidelines (Kilkenny et al., 2010), lack of crucial standards in study design (Moher et al., 2015) such as sample size estimation and blinded assessment of outcome is the primary defect. In future animal studies, the ARRIVE guidelines should be followed; sample size estimations and blinded assessments of outcome should be emphasized. Specific methods of these two points could be referred to in the following two studies (Hayes and Bennett, 1999; Hooijmans et al., 2014). Use of experimental animals with comorbidities such as advanced age, obesity, hypertension, hyperlipidemia, or other risk factors may be more in line with the physiology of diabetic patients and may be helpful for the clinical translation of experimental results (Brosius et al., 2009).

Different animals display varying sensitivities to STZ. C57BL/6 and CD-1 mice are reliably sensitive to STZ (Like and Rossini, 1976; Rossini et al., 1977) as are Wistar and SD rats. Indeed, rats rather than mice tend to be selected to establish diabetic models because their greater size is conducive to monitoring of renal physiology, access to sufficient renal tissue, and repeated blood sampling for analysis. It is difficult but necessary to strictly control the dosage of STZ to avoid problems of high mortality and low modeling rate due to the high sensitivity of rats to STZ. The subgroup analysis indicated that the group in which DN model was induced in the C57BL/6J mice showed greater effect size than that of rats though the dosage of STZ in the mice group (100 mg/kg, IP, QD for 2 d), much larger than that of the rat group (≤65 mg/kg), suggesting that high doses of STZ may cause less *β*-cell damage in mice. Although high-proficiency operational skills and high-quality nursing are required, replacing rats with mice less sensitive to STZ may be beneficial to increase the tolerance to STZ and to reduce the mortality of experimental animals. Furthermore, the included studies used three methods to imitate the characteristics of type 1 diabetes or type 2 diabetes to establish DN model according to varying purposes and laboratory conditions: 18 studies established the DN model using STZ; two used low-dose STZ injection combined with high-fat diet; and four studies used mutant or transgenic mice. In our subgroup analysis, the effect size of AS-IV for decreasing BUN and SCr in various models induced by different methods showed significant differences, suggesting that this factor may be the source of the high degree heterogeneity.

Although no difference was seen between the high- and low-dose AS-IV treatment groups, this subgroup analysis was carried out to determine the sources of high heterogeneity rather than its dose–effect relationship given that it is a comparison under different experimental conditions. We perused all included studies that were designed to study the effects of different doses of AS-IV in DN in the same experiments. Of these, two studies reported that the levels of BUN were markedly greater in the DN-vehicle group than in the control group, both of which were significantly reduced by AS-IV treatment in a dose-dependent manner (3–12 mg/kg). Six studies reported that varying doses of AS-IV (0.75–80mg/kg) decreased the levels of BUN, though not significantly (P > 0.05). Such a large oral dose range yields similar results suggesting that there may be problems in AS-IV absorption or reaction to receptor binding. Actually, previous studies have reported that the high molecular weight and low lipophilicity of AS-IV may limit its passive transport in the intestine which directly results in low permeability and low bioavailability of AS-IV (the absolute bioavailability in rat: 2.2% only) (Gu et al., 2004; Huang et al., 2006). Up to now, various strategies have also been studied to solve this problem. Absorption enhancers of AS-IV that can open the tight junction are one of the major directions to explore the absorption enhance strategy of AS-IV (Gu et al., 2004). Special solvent such as self-microemulsifying drug delivery system was explored to enhance absorption of AS-IV (Zhang et al., 2019). In addition, the development and application of astragaloside injection may effectively solve the intestinal absorption problem of AS-IV (Chen et al., 2019). In the present study, it still stays at the stage to explore whether the drug is effective, and its mechanisms and the results show that AS-IV has renoprotective functions in DN. The relationship between dose and efficacy remains uncertain probably due to low bioavailability of AS-IV at present. Thus, we suggested that the methods of appeal to promote the bioavailability of AS-IV should be applied more in future experiments to explore the optimal dose range of AS-IV in DN. Furthermore, the longer period of AS-IV treatment suggested poorer efficacy than the shorter treatment lasting 8 weeks or less, suggesting that the duration of treatment may be a source of the high degree of heterogeneity. We attribute this to the fact that DN is a progressive and irreversible disease, and extended treatment time of AS-IV merely delayed the progression of DN rather than reversing it.

**Figure 15:**
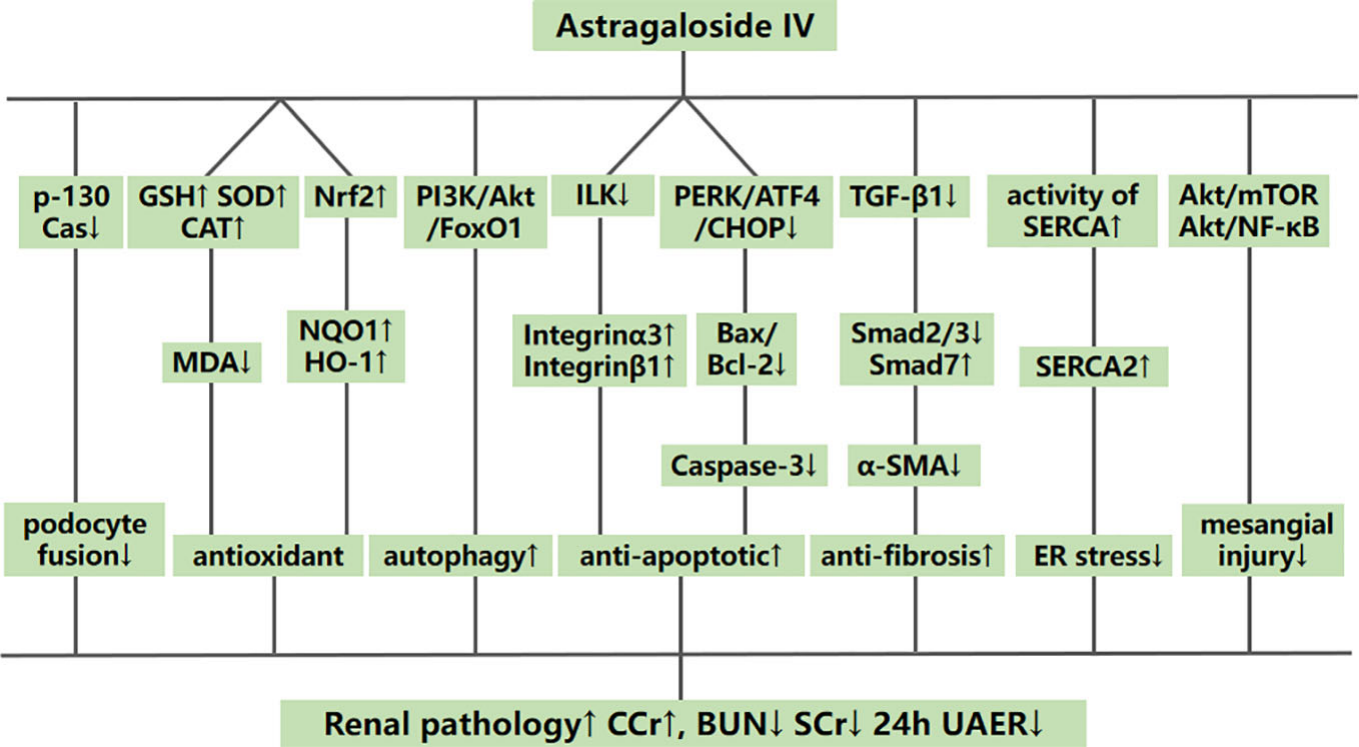
A schematic representation of possible renoprotective mechanisms of astragaloside IV for diabetic nephropathy.

Systematic reviews of preclinical studies are thought to be valuable tools to determine mechanisms and to provide important insights into the designed animal studies (Van et al., 2013). The possible mechanisms of AS-IV that mediated kidney protection in the included studies are summed up as follows: (1) alleviating renal fibrosis by inhibiting the expression of TGF-*β*1 in renal tissues, further decreasing Smad2/3 and enhancing Smad7 levels to reduce the expression of *α*-SMA; (2) antioxidant action by increasing GSH, SOD, and CAT to reduce the release of MDA and enhancing Nrf2 to upregulate the expression of NQO1 and HO-1; (3) inhibiting apoptosis by downregulating the PERK-ATF4-CHOP pathway, Bax/Bcl-2 and the expression of caspase-3; in addition, inhibiting apoptosis in podocytes specifically by downregulating ILK and by upregulating integrins *α*3 and *β*1; by enhancing TUG1 and miR-378 and downregulating the expression of TRAF5; and by up-regulating APN; (4) reducing the proliferation of mesangial cells by regulating the Akt/mTOR pathways; (5) alleviating endoplasmic reticulum stress by enhancing the activity of SERCA and the expression of SERCA2, and reducing the activation of the PERK/eIF2*α* and IRE1/JNK pathways; (6) reducing the damage to mesangial cells by downregulating the Akt/NF-*κ*B pathway; (7) inhibiting mitophagy by downregulating the PINK1/Parkin pathway; (8) inhibiting mitochondrial fission by reducing the expression of renal dynamin-related protein 1 (Drp-1), mitochondrial fission protein 1 (Fis-1), and mitochondrial fission factor (MFF); (9) inhibiting fusion of podocyte processes by downregulating p-130Cas; and (10) increasing autophagic activity of renal tissue cells by downregulating the PI3K/Akt/FoxO1 pathway (**Figure 15**).

## Conclusion

Preclinical *in vivo* evidence suggests that AS-IV has renoprotective functions in DN, probably *via* antifibrotic, antioxidant, and antiapoptotic actions, resulting in alleviation of endoplasmic reticulum stress, inhibition of mitochondrial fission, and increased autophagic activity. Taken together, the findings suggest that AS-IV is a renoprotective candidate drug for treatment of DN.

## Author Contributions

HW, ZZ, and Y-YH contributed equally to this work. QZ, HW, and Y-LW designed the study; HW, YJ, X-JL, and Z-ZZ collected the data; HW, Y-YH, and ZZ performed all analyses. All authors contributed to the article and approved the submitted version.

## Funding

This work was supported by the Clinical Research Foundation of the 2nd Affiliated Hospital of Wenzhou Medical University (SAHoWMU-CR2018-01-105), Lin He’s New Medicine and Clinical Translation Academician Workstation Research Fund (17331208), Wenzhou Science and Technology Bureau Programs (H2015006 and Y20170322), and the Programs of Administration of Traditional Chinese Medicine in Zhejiang (2015ZB077 and 2018ZB080).

## Conflict of Interest

The authors declare that the research was conducted in the absence of any commercial or financial relationships that could be construed as a potential conflict of interest.
